# Idiopathic Intracranial Hypertension in an Adolescent With Recent Human Immunodeficiency Virus (HIV) Diagnosis: A Challenging Etiological Dilemma

**DOI:** 10.7759/cureus.60001

**Published:** 2024-05-09

**Authors:** Imoh L Ebong, Arleen Delgado, Sofia S Aranda, Olufunto O Shonibare, Saman Aryal, Bijaya Karki, Katiusca Acosta

**Affiliations:** 1 Department of Pediatrics, NYC Health+Hospitals/Woodhull Medical Center, New York, USA

**Keywords:** lumbar puncture, body mass index, doxycycline, idiopathic intracranial hypertension, hiv

## Abstract

Idiopathic intracranial hypertension (IIH) or benign intracranial hypertension affects the neuro-ophthalmological system and leads to elevated intracranial pressure. Elevated opening pressure during lumbar puncture is diagnostic of IIH. Here in, we present an interesting case of a 15-year-old girl, recently immigrated and with a high BMI, presenting with recurrent fever, abdominal issues, weight loss, and other symptoms, leading to a diagnosis of pelvic inflammatory disease (PID) and HIV infection. After treatment with antibiotics (doxycycline) and antiretroviral therapy, she developed IIH, manifesting as sudden-onset headache and vision problems. MRI and lumbar puncture confirmed the diagnosis. She responded well to acetazolamide and was discharged with continued medication and follow-up appointments. This case underscores the complexity of IIH development, especially in the setting of acute HIV infection and antibiotic treatment, highlighting the need for a comprehensive diagnostic approach and multidisciplinary management.

## Introduction

Idiopathic intracranial hypertension (IIH) is a medical condition that affects the neuro-ophthalmological system and causes an elevation in pressure within the skull. This medical condition is also known as pseudotumor cerebri or benign intracranial hypertension. The diagnosis of IIH is given when no pathological abnormalities are detected on neuroimaging and frequently observable signs of increased intracranial pressure (ICP) are present [1].

IIH is diagnosed using a lumbar puncture (LP) to measure the opening pressure (OP) of the cerebrospinal fluid (CSF). If the CSF composition is normal and the OP is calculated to be 25cm H2O or higher, it may indicate IIH. Sixth cranial nerve palsy and papilledema are hallmarks of IIH, and neurological examinations should be unremarkable except for these. Ophthalmological examinations may also be conducted to confirm and evaluate the degree of papilledema [2].

The CDC reported in 2019 that adolescents and young adults accounted for 21% of HIV diagnoses in the United States. Regrettably, this age group has the slightest awareness of their HIV status, the lowest rates of testing and recent infection, and the lowest percentage of individuals in care or with a suppressed viral load [3].

ICP may develop during various phases of HIV infection, mainly in advanced stages caused by opportunistic infections like cryptococcus meningitis, tuberculosis, and syphilis or from neoplasms. However, ICP seldom arises in the initial stages of the disease, either during lymphocytic meningitis acute HIV infection (AHI) or as a trigger for IIH [4-6].

Certain medications, such as tetracyclines like minocycline and doxycycline, steroids, nalidixic acid, and amiodarone, can cause intracranial hypertension. While the exact mechanism for these reactions is not known, several reports have linked drugs like doxycycline to increased ICP. There are various proposed mechanisms for how tetracyclines can cause intracranial hypertension, such as interfering with the energy-dependent absorption mechanism by affecting cyclic adenosine monophosphate at the arachnoid granulations [7,8].

In our case, there were several risk factors associated with the development of IIH, including female gender within the reproductive age group, obesity, recent doxycycline treatment, and HIV infection. However, it is challenging to attribute the emergence of IIH solely to any specific factor, as the condition can be multifactorial.

## Case presentation

This is a 15-year-old female recent immigrant, newly diagnosed with HIV infection following complaints of oral thrush, maculopapular rash and symptoms suggestive of pelvic inflammatory disease (PID), on oral doxycycline and metronidazole for the treatment of PID and nystatin for oral thrush. She returned to the emergency room with complaints of headaches, blurred vision, and right eye deviation for two days. The headaches were described as sudden in onset, localized to the temporal region bilaterally, and radiated to the occiput, rated a 9/10 in severity, persistent, and worst in the supine position. The headaches were associated with bilateral blurred vision, diplopia, photophobia, and dizziness. Further review of symptoms was negative for nausea, vomiting, prior trauma to the head or fall, numbness, weakness, seizures, loss of consciousness, pain with eye movement, or the use of corrective lenses. 

Physical examination was remarkable for BMI in the 90th percentile and slight medial deviation of the right eye. Neurological examination was notable for CN III, IV, and VI deficits (esotropic bilateral abduction deficit (OD>OS)). There were no signs of meningitis and gait was normal. Visual acuity was 20/20 in the left eye and 20/40 in the right eye, and grade 1-2 bilateral papilledema was noted.

Her blood tests and culture results are shown in Table 1 below.

**Table 1:** Blood and Culture Test Results HCV: hepatitis C virus, IgG: immunoglobulin G, Ab: antibodies, Ag: antigen

Test	Result	Reference Range
WBC	5.07	4.5 - 13 x 10(3)/mcL
Hemoglobin	11.7	12 - 16 g/dL
Platelet	420	150- 440 x 10(3)/mcL
HIV-1 Viral Load	3,160.000	<=20 copies/mL
T-cell subset ABS CD3-	986	1000 - 2200 cells/uL
Toxoplasma IgG Screen	< 3	<=7.1 IU/mL
Lead	<1	0.0 - 3.4 ug/dL
Hemoglobin A1C	6	<= 5.6 %
HCV	Weakly reactive	-
Hepatitis C RNA Qual	Negative	-
Hepatitis B surface Ag	Nonreactive	-
Hepatitis B surface Ab	Nonreactive	-
Treponema Pallidum Ab Screen I	Negative	-
Cryptococcal Ag Serum	Negative	-
Urine culture	No growth	-
Chlamydia (urine)	Negative	-
Gonorrhea (urine)	Negative	-
Strongyloides Antibodies	Negative	-

**Figure 1:**
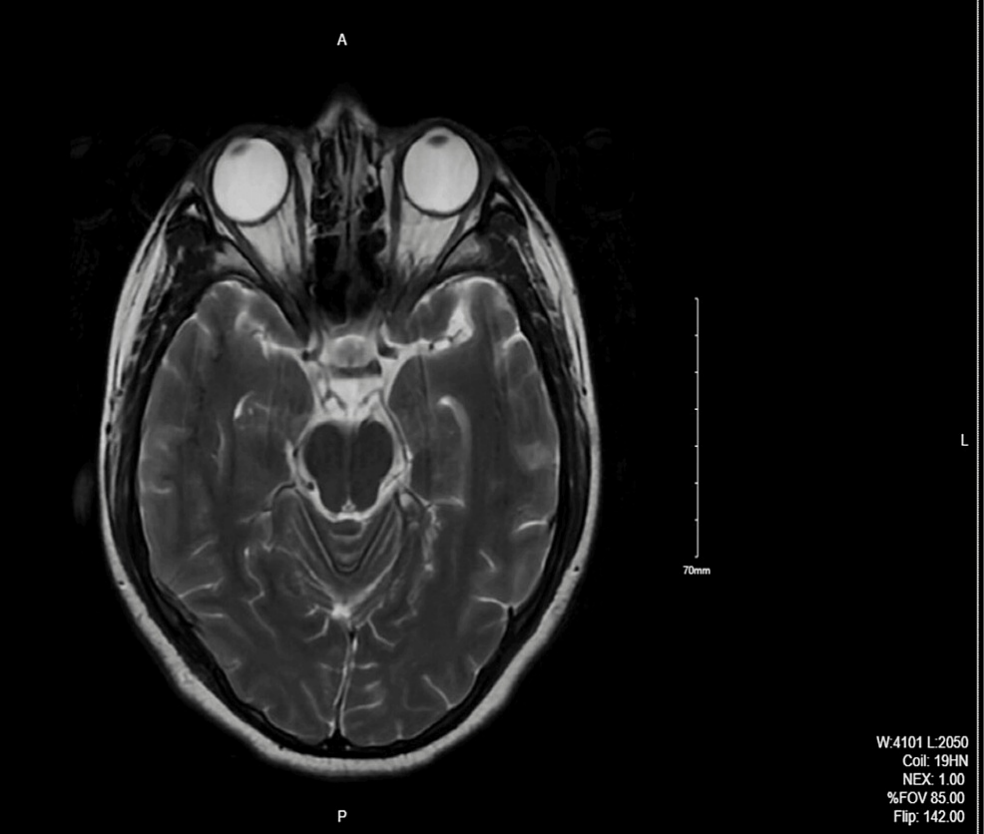
MRI of the orbits with contrast: The globes show mild protrusion of the optic discs and flattening of the posterior sclera bilaterally.

**Figure 2:**
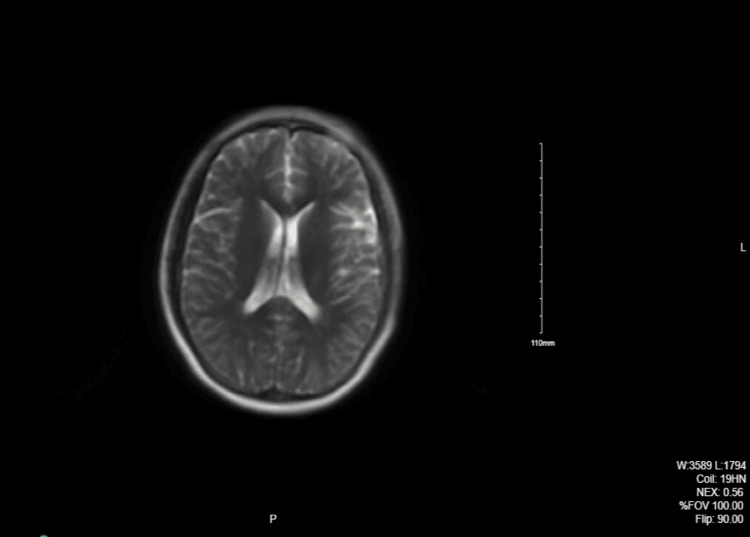
MRI of the brain with contrast: Normal study.

MRI of the orbits showed mild protrusion of the optic discs and flattening of the posterior sclera bilaterally which was suggestive of increased ICP. Head CT scan and Brain MRI were normal (Figures 1, 2).

LP was remarkable for increased opening pressure (36 cm H2O). CSF studies were remarkable for protein and negative for all infectious markers.

**Table 2:** Cerebrospinal Fluid (CSF) Analysis and Culture Results PCR: polymerase chain reaction, IgG: immunoglobulin G, IgM: immunoglobulin M, Ag: antigen

Test	Result	Reference Range
CSF Glucose	57	40- 70 mg/dL
CSF Protein	69	10 – 40 mg/dL
WBC	20	0-5 cells/ mcL
RBC	0	0-3 cells/ mcL
Herpes simplex 1/2 CSF PCR	Negative	-
Cryptococcal Ag CSF	Negative	-
CSF culture	No growth	-
Cryptococcal Ag CSF	Non-Reactive	-
Toxoplasmosis- CSF IgG	< 0.90	< 0.90
Toxoplasmosis-CSF IgM	< 0.80	< 0.80
Fungus CSF/ India Ink	Negative	-
BioFire Meningitis Encephalitis Panel	Negative	-

Due to the grade 1-2 bilateral papilledema, the ophthalmologist in consultation with a pediatric neurologist started the patient on acetazolamide to help decrease ICP. She was also started on acyclovir, cefepime, and vancomycin for meningitis coverage pending lumbar puncture and CSF studies, which were discontinued following negative CSF culture results. 

Resolution of symptoms was noted following the lumbar puncture, including improvement of her visual acuity to 20/20 bilaterally. The patient was discharged home on acetazolamide (for elevated ICP), Biktarvy (for HIV treatment), and metronidazole (to complete PID treatment) and follow-up appointments were scheduled with Infectious Diseases, Neurology, Ophthalmology, and Primary Care Clinic.

## Discussion

Idiopathic intracranial hypertension, also known as pseudotumor cerebri, manifests as an increased intracranial pressure in patients devoid of structural brain or CSF abnormalities, and it is typically a diagnosis of exclusion. The condition exhibits a demographic preference for females, with an observed correlation to elevated BMI, the underlying reasons for which remain poorly understood. 

The classical presentation of IIH in patients with HIV infection can vary. However, individuals with HIV who develop IIH typically present with symptoms similar to those seen in non-HIV-infected individuals. It's important to note that HIV infection can complicate the clinical picture, potentially leading to atypical presentations (aseptic meningitis features, headache as the only cardinal symptom) or additional symptoms related to the infection or associated conditions [9-12]. Thus, careful evaluation and monitoring are essential in patients with HIV who present with symptoms suggestive of IIH. 

Our patient, a young, overweight female (BMI 93rd percentile for age) fits the typical demographic and presents with classical symptoms of IIH, including headaches, visual disturbances such as blurry vision, and diplopia, in the presence of papilledema. Additionally, there was evidence of cranial nerve palsy, attributed to unilateral compromise of the abducens nerve (VI).

Given the coexistence of HIV diagnosis, it becomes imperative to rule out opportunistic infectious pathogens, such as Cryptococcus neoformans or Toxoplasma, and lymphoma. These possibilities were deemed unlikely, considering the patient's immunological status, reflected by a CD4 count of 357, and were further excluded through unremarkable cerebrospinal fluid analysis, head CT scan, and brain MRI.

HIV infection, at various stages of the disease, has been previously associated with secondary causes of intracranial hypertension [9-12], along with other neurological complications like Guillain-Barré syndrome and neurocognitive disorders, even in the absence of immunological deficiency [13]. Neurotropism of HIV has been proven by findings of the virus in the nervous system as in microglial cells and astrocytes [6].

It is also interesting that she developed raised ICP after she was discharged home on doxycycline to complete her treatment for PID. Literature has shown that doxycycline has also been implicated in the etiology of raised ICP. With our patient, it is sort of a gray area to clearly distinguish what her symptoms may have been specifically due to the fact that HIV infection has an association with raised ICP. Treatment of idiopathic intracranial hypertension focuses on relieving symptoms, reducing the intracranial pressure, and preserving vision.

Following LP, her symptoms resolved, she was also on acetazolamide which helped reverse symptoms. In the absence of a preexisting vision screen result being that she was a recent immigrant with no pre-existing records, it may be difficult to tell if the 20/40 acuity in the left eye was from her condition. Follow-up with ophthalmology showed 20/20 visual acuity subsequently.

Furthermore, with doxycycline-induced IIH, most patients have a resolution of their symptoms when the medication is discontinued usually because the half-life is less than 24 hours. Our patient was placed on another medication to complete the treatment of PID. In a series of 12 patients who had minocycline-associated raised ICP, there was no documented recurrence following cessation of the medication and patients had visual acuity back to 20/20 or even better [6]. Minocycline, doxycycline, and tetracycline have the propensity to cause raised ICP with minocycline being a worse culprit due to its increasing CSF penetration comparatively. 

Despite pointers favoring doxycycline as the cause of the raised ICP in our patient, it is also pertinent to consider her HIV status. Since both are connected to her diagnosis, it is important to consider either or in making a definite diagnosis.

## Conclusions

In conclusion, our case emphasizes the diagnostic complexities associated with IIH, particularly when occurring alongside HIV infection. The patient's alignment with IIH demographics, combined with classical symptoms and neurological manifestations, underscores the necessity for a thorough diagnostic approach. Comprehensive investigations excluded opportunistic infections and lymphoma, supporting the primary diagnosis of IIH. The intriguing link between HIV and secondary intracranial hypertension prompts further exploration of the virus's neurotropism and its impact on neurological complications. This case highlights the significance of considering both primary and secondary etiologies in complex medical histories, revealing the intricate interplay between neurological manifestations and systemic conditions. Further research is crucial for a deeper understanding of these associations.
